# Oncogene- and Oxidative Stress-Induced Cellular Senescence Shows Distinct Expression Patterns of Proinflammatory Cytokines in Vascular Endothelial Cells

**DOI:** 10.1155/2013/754735

**Published:** 2013-09-30

**Authors:** Etsu Suzuki, Masao Takahashi, Shigeyoshi Oba, Hiroaki Nishimatsu

**Affiliations:** ^1^Institute of Medical Science, St. Marianna University School of Medicine, 2-16-1 Sugao, Miyamae-ku, Kawasaki 216-8512, Japan; ^2^The Department of Internal Medicine, Faculty of Medicine, University of Tokyo, 7-3-1 Hongo, Bunkyo-ku, Tokyo 113-8655, Japan; ^3^The Department of Urology, Faculty of Medicine, University of Tokyo, 7-3-1 Hongo, Bunkyo-ku, Tokyo 113-8655, Japan

## Abstract

Senescent cells are metabolically active and produce a variety of proinflammatory cytokines. It was previously reported that atherosclerotic plaques contain senescent cells, suggesting that senescence may contribute to the progression of atherosclerosis. In this study, we induced cellular senescence in vascular endothelial cells (VECs) using hydrogen peroxide (H_2_O_2_) or an adenovirus that expresses a constitutively active mutant of Ras (AdRas12V) and studied the expression of cytokines. Both H_2_O_2_ treatment and AdRas12V infection induced senescence in VECs, as assessed by senescence-associated *β*-Gal activity and the expression of proteins such as p53 and p21^CIP1^. In addition, both treatments induced the expression of a variety of cytokines, including interleukin-1*β* (IL-1*β*) and nerve growth factor (NGF). AdRas12V infection induced IL-1*β* expression more significantly than H_2_O_2_ treatment, whereas both treatments induced comparable mRNA and protein expression levels of NGF. These results suggest that senescent cells express different patterns of proinflammatory cytokines, depending on the trigger that induced senescence. It is therefore possible that senescent cells can differentially induce inflammation in the surrounding tissues, depending on the cause of senescence.

## 1. Introduction

 It is well established that primary cultured cells that have been explanted from tissues do not proliferate indefinitely and eventually exit the cell cycle. Hayflick observed this phenomenon and hypothesized that the finite lifecycle of these cells may be an expression of aging or senescence at the cellular level [1]. Cellular senescence therefore represents a stable and long-term cell cycle arrest, although cells remain viable and metabolically active. Senescent cells produce a variety of cytokines and chemokines, which may explain why senescent cells cause inflammation in the surrounding tissues [2]. Although telomere shortening is a major cause of cellular senescence (known as replicative cellular senescence) [3], other stimuli, such as the activation of oncogenes and oxidative stress, can also cause cellular senescence (termed premature cellular senescence) [4, 5]. When cells are damaged, they withdraw from the cell cycle and try to repair the damage by activating the p53-p21^CIP1^ and retinoblastoma protein (pRb)-p16^INK4A^ pathways. The activation of these pathways is therefore an important factor during cellular senescence [2, 6]. 

 Senescent cells exhibit a significantly altered morphology. They become large, flat, and multinucleated. They sometimes become spindle-shaped, depending on the senescence trigger. Although there is no single marker by which senescent cells are identified, senescence-associated (SA)-*β*-Galactosidase (*β*-Gal) activity is observed in senescent cells [7]. Senescent cells also express p53, the hypophosphorylated (active) form of pRb, and cyclin-dependent kinase (cdk) inhibitors such as p16^INK4A^ and p21^CIP1^ [2, 6], because senescent cells have withdrawn from the cell cycle.

 It has recently been shown that human atherosclerotic plaques contain SA-*β*-Gal positive vascular endothelial cells (VECs) and vascular smooth muscle cells (VSMCs) that exhibit the morphological features of senescence [8, 9]. It has also been reported that interleukin-1*β* (IL-1*β*) is expressed in senescent cells located in human atherosclerotic lesions [8], suggesting that senescent cells may promote inflammation in the lesions. Although several different pathways such as telomere shortening, oncogene activation, and oxidative stress induce a common phenotype of senescence, it remains unclear whether senescent cells show the same patterns of cytokines expression, regardless of the trigger. A distinct pattern of cytokine production may be stimulated depending upon the nature of inducer, thereby causing different patterns of inflammation in surrounding tissues.

 In this study, we used a constitutively active mutant of Ras to induce oncogene-induced senescence and hydrogen peroxide (H_2_O_2_) to induce oxidative stress-induced senescence in human umbilical vein endothelial cells (HUVECs). We examined whether these two pathways induce different patterns of cytokines expression.

## 2. Materials and Methods

### 2.1. Reagents

Anti-p21^CIP1^, anti-NF-*κ*B p65, and anti-*β*-actin antibodies were purchased from Santa-Cruz Biotechnology, Inc. (Santa-Cruz, CA), and anti-p53 antibody was obtained from Abcam (Tokyo, Japan). Anti-phospho-NF-*κ*B p65 (Ser536) antibody, which recognizes the catalytically active form of the p65 subunit of NF-*κ*B, was obtained from Cell Signaling Technology, Inc. (Danvers, MA). BAY11-7082 was purchased from Sigma-Aldrich (St. Louis, MO).

### 2.2. Cell Culture

HUVECs were purchased from Sanko Junyaku Co., Ltd. (Tokyo, Japan) and cultured in HuMedia-EG2 (Kurabo, Osaka, Japan). To induce senescence by treatment with H_2_O_2_, HUVECs were incubated with 100 *μ*mol/L H_2_O_2_ for 1 hr. After washing with phosphate-buffered saline (PBS), HUVECs were cultured in HuMedia-EG2 for 7 days.

### 2.3. Adenoviral Infection

Infection with a replication-defective adenovirus that expresses a constitutively active mutant of mouse Ras (AdRas12V) was reported previously [10]. A recombinant adenovirus that expresses green fluorescence protein (AdGFP) was obtained from Quantum Biotechnologies (Montreal, Canada). HUVECs were infected with these adenoviruses at a multiplicity of infection (MOI) of 20, and then were cultured in HuMedia-EG2 for 7 days to induce senescence.

### 2.4. SA-*β*-Gal Assay

SA-*β*-Gal staining was performed using a cellular senescence detection kit (Cell Biolabs, Inc., San Diego, CA) according to the manufacturer's protocol.

### 2.5. RNA Extraction and Real Time PCR Analysis

Total RNA was extracted using TRIzol reagent (Gibco-BRL, Rockville, MD) according to the manufacturer's instructions. Total RNA was reverse transcribed using a ReverTra Ace qPCR RT Kit (TOYOBO, Osaka, Japan). The expression of human IL-1*β*, interleukin-6 (IL-6), nerve growth factor (NGF), and glyceraldehyde 3-phosphate dehydrogenase (GAPDH) was examined by real time PCR using SYBR Green (Thunderbird SYBR qPCR Mix, TOYOBO, Japan). The following primers were used:

 IL-1*β*sense: 5′-CGAATCTCCGACCACCACTAC-3′,
 IL-1*β*antisense: 5′-TCCATGGCCACAACAACTGA-3′,
 IL-6sense: 5′-TAGCCGCCCCACACAGA-3′,
 IL-6antisense: 5′-TCGAGGATGTACCGAATTTGTTT-3′,
 NGFsense: 5′-GGGCGAATTCTCGGTGTGT-3′,
 NGFantisense: 5′-TGTCTGTGGCGGTGGTCTTA-3′,
 GAPDHsense: 5′-ACCCACTCCTCCACCTTTGA-3′,
 GAPDHantisense: 5′-CATACCAGGAAATGAGCTTGACAA-3′.

Real time PCR was performed using an ABI PRISM 7000 sequence detection system (Applied Biosystems, Foster City, CA). To confirm that no significant quantities of primer dimers were formed, dissociation curves were analyzed.

### 2.6. Protein Extraction and Western Blot Analysis

Proteins were extracted from HUVECs in a cell lysis buffer (50 mmol/L Tris-HCl (pH 8.0), 150 mmol/L NaCl, 1% NP-40) containing 2 *μ*g/mL aprotinin, 2 *μ*g/mL leupeptin, and 1 mmol/L phenylmethylsulfonyl fluoride. Western blot analysis was performed as previously described [11].

### 2.7. Enzyme-Linked Immunosorbent Assay

Human IL-1*β* and NGF in culture media were measured with enzyme-linked immunosorbent assay (ELISA) kits (Abcam, Tokyo, Japan) according to the manufacturer's instructions. 

### 2.8. Statistical Analyses

All values are expressed as the mean ± SEM. Statistical analyses were performed using analysis of variance followed by the Student-Neumann-Keuls test. Differences with a *P* value of <0.05 were considered to be statistically significant.

## 3. Results and Discussion

### 3.1. Oncogene Activation and Oxidative Stress Induce Cellular Senescence in HUVECs

**Figure 1:**
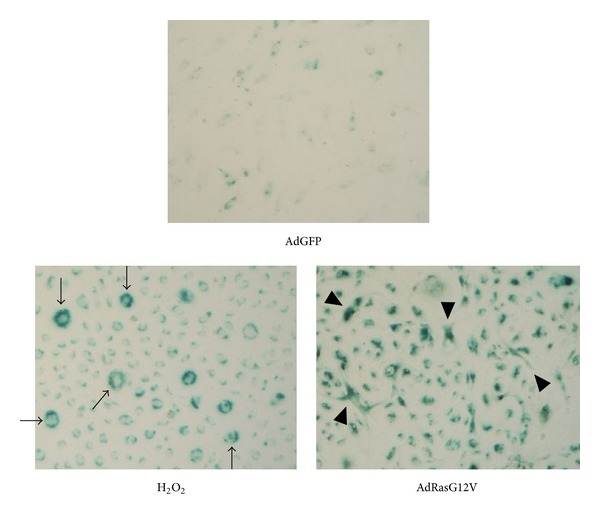
SA-*β*-Gal staining of HUVECs. HUVECs were infected with AdRas12V for 1 week to induce senescence or with AdGFP as the negative control. Senescence was also induced in HUVECs treated with 100 *μ*mol/L H_2_O_2_ for 1 hr. Arrows indicate large and round-shaped senescent cells, and arrowheads indicate flat or spindle-shaped senescent cells.

**Figure 2:**
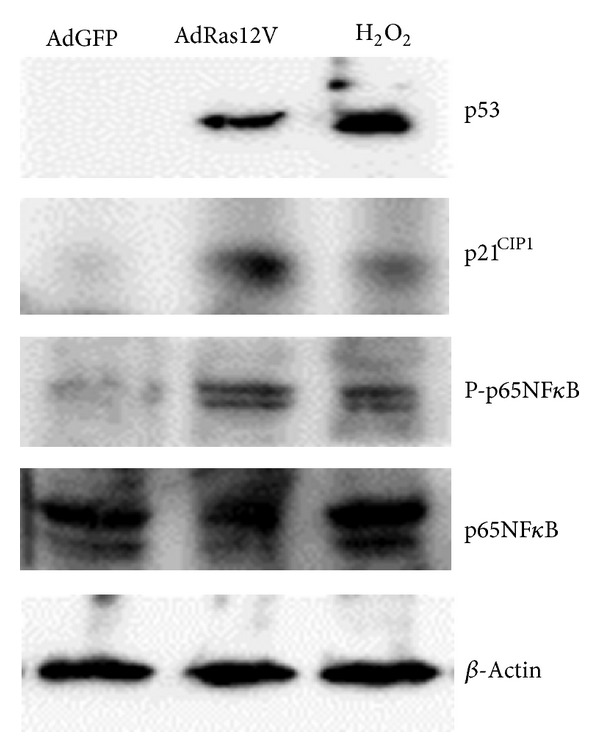
Western blot analysis of proteins extracted from HUVECs. HUVECs were infected with AdRas12V or treated with H_2_O_2_. Proteins were extracted from HUVECs 1 week after the treatment and analyzed by western blot analysis.

 We first examined whether oncogene activation and oxidative stress induced cellular senescence in HUVECs by SA-*β*-Gal staining (Figure 1). HUVECs pretreated with H_2_O_2_ or infected with AdRas12V were positively stained in contrast to noninfected control cells (data not shown) or those infected with AdGFP, suggesting that both treatments induced senescence in HUVECs. Because there were no significant differences in SA-*β*-Gal staining between noninfected HUVECs and AdGFP-infected HUVECs, we used AdGFP-infected HUVECs as the negative control throughout this study. Interestingly, although both H_2_O_2_ treatment and AdRas12V infection induced SA-*β*-Gal positive cells, morphology of the cells was apparently different. While cells treated with H_2_O_2_ were large and round-shaped, those infected with AdRas12V became spindle-shaped or flat. This suggests that the senescent cells may have different functions, depending on the trigger that induced senescence. We also performed western blot analysis to confirm that these treatments induced senescence (Figure 2). The expression of p53 in HUVECs treated with H_2_O_2_ or infected with AdRas12V was significantly higher than that in control cells, suggesting that the DNA-damage response had been activated. Both treatments also stimulated the expression of the cdk inhibitor p21^CIP1^, suggesting that the cells had withdrawn from the cell cycle. It has been reported that NF-*κ*B is activated in senescent cells, which helps maintain the senescent phenotype and stimulates the production of cytokines [12, 13]. We, therefore, examined whether NF-*κ*B was activated in our system. The p65 subunit of NF-*κ*B is reportedly phosphorylated at Serine-536 when NF-*κ*B is activated. Both H_2_O_2_ treatment and AdRas12V infection stimulated the phosphorylation of p65 at Serine-536, suggesting that both treatments activated NF-*κ*B.

### 3.2. Cytokine Production in Senescent Cells

**Figure 3:**
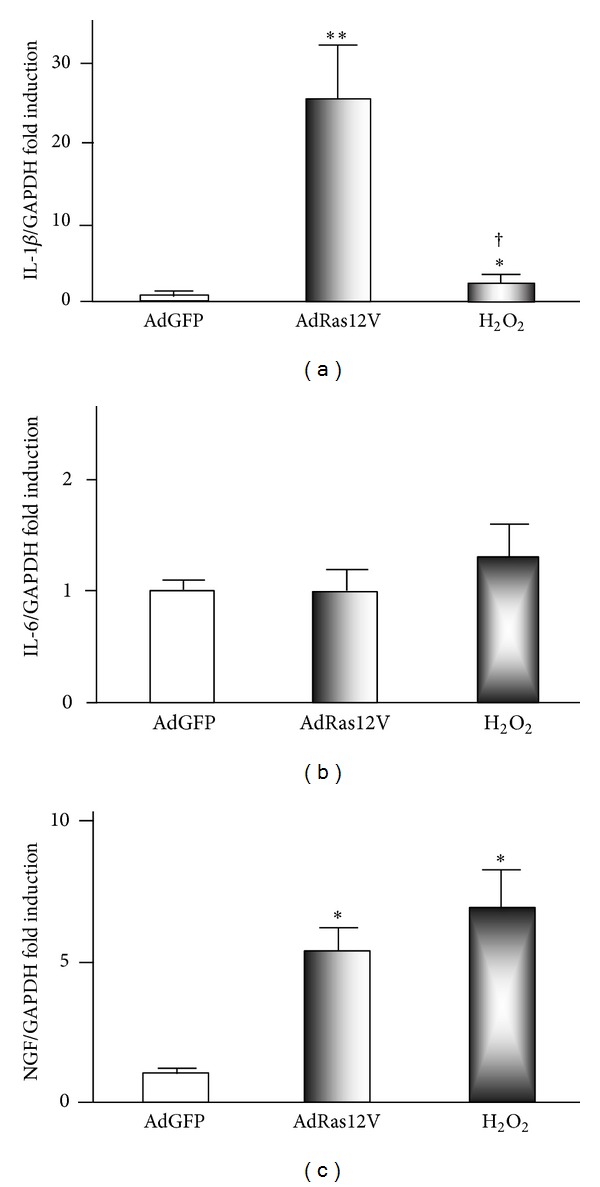
Real time PCR analysis of the expression of proinflammatory cytokines in HUVECs. HUVECs were infected with AdGFP or AdRas12V or treated with 100 *μ*mol/L H_2_O_2_. After 1 week, total RNA was extracted for real time PCR analysis. The expression of IL1-*β*, IL-6, and NGF was normalized to GAPDH. ∗ and ∗∗: *P* < 0.05 and *P* < 0.01, respectively, versus AdGFP infection. †: *P* < 0.01 versus AdRas12V infection (*n* = 6 per group).

**Figure 4:**
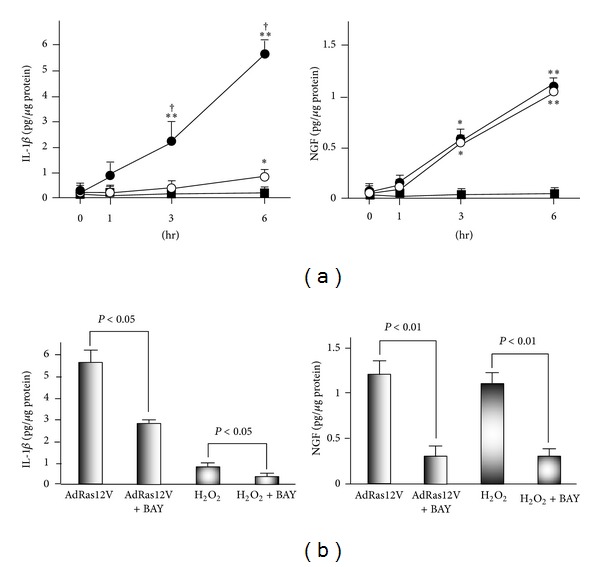
The expression of IL-1*β* and NGF proteins in HUVECs treated with AdRas12V or H_2_O_2_. (a) Time course of the accumulation of IL-1*β* and NGF in the culture media. HUVECs were infected with AdGFP (closed squares) or AdRas12V (closed circles) or treated with H_2_O_2_ (open circles). After 1 week, HUVECs were washed with PBS, and medium was replaced with serum free Dulbecco's modified Eagle medium (DMEM). HUVECs were incubated for the indicated periods, and culture media were collected for ELISA. ∗ and ∗∗: *P* < 0.05 and *P* < 0.01, respectively, versus AdGFP infection at each time point. †: *P* < 0.05 versus H_2_O_2_ treatment at each time point (*n* = 6 per group). (b) The effect of BAY11-7082 on cytokine production. Experiments were performed as in (a). In addition, some HUVECs were preincubated with 10 *μ*mol/L of BAY11-7082 (Bay) for 2 hrs and then washed with PBS. The medium was replaced with serum free DMEM. HUVECs were then incubated for 6 hrs in the presence or absence of Bay (*n* = 6 per group).

 It is well established that senescent cells undergo massive changes in gene expression and actively produce a variety of proinflammatory cytokines and immune modulators. These changes have been referred to as the senescence-associated secretory phenotype [14]. We used real time PCR to examine whether H_2_O_2_ treatment and AdRas12V infection induced similar patterns of cytokine production (Figure 3). Although both treatments significantly stimulated IL-1*β* expression compared with AdGFP infection, the levels induced by AdRas12V were significantly higher than those induced by H_2_O_2_. IL-6 is a well-known cytokine that senescent cells produce [15, 16]. However, expression of IL-6 in AdRas12V-infected or H_2_O_2_-treated HUVECs was similar to that in AdGFP-infected HUVECs. The expression of NGF in cells treated with H_2_O_2_ or infected with AdRas12V was significantly higher than that in AdGFP infection, and the expression in the two treatment groups was similar. In addition, we assessed the expression of several other cytokines, including tumor necrosis factor-*α* and vascular endothelial growth factor-A. Although both treatments significantly induced the expression of these cytokines, the extent (up to 2-fold induction) was smaller than that observed in NGF expression (data not shown). We further examined the expression of IL-1*β* and NGF at the protein level by ELISA (Figure 4(a)). H_2_O_2_ treatment and AdRas12V infection increased the accumulation of IL-1*β* in the culture medium in a time-dependent manner. AdRas12V infection increased the accumulation of IL-1*β* in the culture medium more significantly than H_2_O_2_ treatment. H_2_O_2_ treatment and AdRas12V infection also increased the accumulation of NGF in a time-dependent fashion, and both treatments had a similar effect. To study the role of NF-*κ*B in cytokine production, we examined the effects of BAY11-7082, an I*κ*B*α* inhibitor, on IL-1*β* and NGF production (Figure 4(b)). Pretreatment with BAY11-7082 significantly inhibited the secretion of IL-1*β* and NGF induced by H_2_O_2_ treatment and AdRas12V infection.

 We found that senescent HUVECs produced a variety of proinflammatory cytokines and that the production was mediated, at least in part, by the NF-*κ*B-dependent pathway. Thus, the NF-*κ*B-dependent pathway appears to play a pivotal role in the production of cytokines in senescent VECs. We also found that although H_2_O_2_ treatment and AdRas12V infection significantly induced IL-1*β* expression, AdRas12V infection had a more significant effect than H_2_O_2_ treatment. In contrast, both treatments had a comparable effect on NGF expression. IL-1*β* is a potent proinflammatory cytokine that stimulates the expression of adhesion molecules on endothelial cells [17] and is frequently expressed in human atherosclerotic plaques [8]. In addition, IL-1*β* deficiency decreases the severity of atherosclerosis in apolipoprotein E (apoE) knock-out mice [18]. IL-1*β* therefore potently induces vascular inflammation. NGF is a member of the neurotrophin family that stimulates not only nerve growth and survival but also mast cell accumulation in various organs [19]. NGF can also promote angiogenesis [20]. Although the role of NGF in atherosclerosis has not been fully elucidated, evidence suggests that it plays a role in the progression of atherosclerosis. The expression of NGF was increased in injured arteries after arterial balloon injury [21]. NGF has a potential to stimulate the migration of VSMCs [21, 22]. The production of IL-1*β* and NGF by senescent cells therefore has the potential to stimulate vascular inflammation. Because AdRas12V infection induced higher levels of IL-1*β* expression than H_2_O_2_ treatment, senescent cells may differentially stimulate inflammation in the surrounding tissues, depending on the senescence trigger. However, future studies are required to expand these observations.

## 4. Conclusions

 Oxidative stress and oncogene activation induced senescence in VECs, and both stimuli promoted the production of proinflammatory cytokines. However, oncogene activation stimulated production of IL-1*β* more significantly than oxidative stress. These data suggest that senescent cells may have different proinflammatory actions on surrounding tissues, depending on the senescence trigger.
